# Development of Graphene Nano-Platelet Based Counter Electrodes for Solar Cells

**DOI:** 10.3390/ma8095284

**Published:** 2015-09-07

**Authors:** Iftikhar Ahmad, Joseph E. McCarthy, Alexander Baranov, Yurii K. Gun’ko

**Affiliations:** 1 School of Chemistry and CRANN, Trinity College Dublin, Dublin 2, Ireland; E-Mails: iftikhaa@tcd.ie (I.A.); mccartj5@tcd.ie (J.E.M.); 2 International Research and Education Centre for Physics of Nanostructures, ITMO University, St. Petersburg 197101, Russia; E-Mail: a_v_baranov@mail.ifmo.ru

**Keywords:** graphene, nano-platelets, counter electrodes, solar cells, DSSC

## Abstract

Graphene has been envisaged as a highly promising material for various field emission devices, supercapacitors, photocatalysts, sensors, electroanalytical systems, fuel cells and photovoltaics. The main goal of our work is to develop new Pt and transparent conductive oxide (TCO) free graphene based counter electrodes (CEs) for dye sensitized solar cells (DSSCs). We have prepared new composites which are based on graphene nano-platelets (GNPs) and conductive polymers such as poly (3,4-ethylenedioxythiophene) poly(styrenesulfonate) (PEDOT:PSS). Films of these composites were deposited on non-conductive pristine glass substrates and used as CEs for DSSCs which were fabricated by the “open cell” approach. The electrical conductivity studies have clearly demonstrated that the addition of GNPs into PEDOT:PSS films resulted in a significant increase of the electrical conductivity of the composites. The highest solar energy conversion efficiency was achieved for CEs comprising of GNPs with the highest conductivity (190 S/cm) and n-Methyl-2-pyrrolidone (NMP) treated PEDOT:PSS in a composite film. The performance of this cell (4.29% efficiency) compares very favorably to a DSSC with a standard commercially available Pt and TCO based CE (4.72% efficiency in the same type of open DSSC) and is a promising replacement material for the conventional Pt and TCO based CE in DSSCs.

## 1. Introduction

Dye-sensitized Solar Cells (DSSCs) have been intensively studied due to their potentially lower costs and easier manufacturing process compared to silicon based solar cells [1,2]. The platinum (Pt) deposited on a transparent conductive oxide (TCO) substrate [3] is widely used as a standard counter electrode (CE) for DSSCs [4]. However, the Pt coated TCO-CE is the most expensive component in a DSSC [5,6] and its high cost is one of the factors limiting the mass production of DSSCs. Therefore, it is highly important to develop a cost-effective counter electrode without the use of Pt and costly TCOs for use in DSSCs [7]. This requires new low-cost CE materials that are simultaneously abundant, non-toxic and have the ability to provide high power conversion efficiencies in DSSCs. New electrode materials and technology should also have the potential for scale up in an industrial production set up. To provide good cell power conversion efficiency a CE should have high electrical conductivity and high electrocatalytic activity towards the iodide/tri-iodide redox reaction (I^−^/I^−^_3_). Furthermore, these CE materials should be chemically stable in the highly reactive electrolyte systems used in DSSCs.

There are various types of materials (e.g., carbon black, carbon nanotubes, graphene, nitrogen-doped graphene nanoribbons, NiS_2_ nanocrystals, nickel nanoparticles, cobalt sulfide nanoparticles, graphene—conductive polymer composites, *etc*.) that have been used to replace Pt in CEs for DSSCs [8,9,10,11,12,13,14,15,16,17,18,19,20,21,22,23,24,25,26,27,28,29]. However, there are only a few reports on a complete replacement of Pt as well as the TCO of the CE in the DSSC with various materials [7,21,30]. For example, Veerappan *et al*., used a graphite film to replace Pt and FTO materials in the CE. They have demonstrated power conversion efficiencies of 5.0% and 6.8% with a graphite/glass based CE and with the standard Pt/FTO/glass-CE/DSSCs respectively [31]. Lee *et al*., employed the conducting polymer PEDOT as a Pt and FTO free CE, achieving power conversion efficiencies of 5.08% and 5.88% with a PEDOT/glass-CE and Pt/FTO/glass-CE/DSSCs respectively [30]. Similarly, Zhang *et al*., reported a TCO and Pt-free CE, which is based on a PEDOT:PSS film as a conductive substrate and an electro- deposited PEDOT layer as the electrocatalytic layer (PEDOT/PEDOT:PSS/glass). DSSCs with this electrode showed a high power conversion efficiency of 8.33%, which was comparable to that of the standard Pt/FTO/glass-CE/DSSC (8.75%) [32]. More recently Chung *et al*., reported a Pt and TCO free CE/DSSC that displayed power conversion efficiencies of 4.1% and 5.6% with glycerol doped PEDOT:PSS and ultraviolet oxidized MWCNT/PEDOT:PSS films respectively [33].

Jiang *et al*. [34] reported a graphene and Pt/graphene based TCO and Pt free CE/DSSCs, showing power conversion efficiencies of 2.16% with graphene/glass and 6.09% with Pt/graphene/glass compared to 5.76% with the standard Pt/FTO/glass-CE/DSSCs. These studies show that graphene based films have sufficient electrical conductivity to replace the TCO of the CE of the DSSC but its electrocatalytic activity is lower than a CE employing Pt.

Polymer materials have found a number of important applications in DSSCs as luminescent and protective coatings [35], templates for designing new nanostructured TiO_2_ electrodes [36], stable electrolytes [37,38,39], conductive plastic substrates [40], counter electrodes [41] and other components of solar cells [42,43,44].

In this paper we report the development of new Pt and TCO free CEs which are based on different types of commercially available graphene nano-platelets (GNPs) combined with conductive PEDOT:PSS polymer composition and the fabrication and testing of the CEs in DSSCs. We also performed detailed investigations of new GNPs based electrodes by Raman spectroscopy, electron microscopy, thermogravimetric analysis and electrical conductivity measurements. We have also studied the effect sintering of the GNP—polymer composites has on their photovoltaic performance in DSSCs.

## 2. Results and Discussion

### 2.1. Preparation and Characterisation of Pt and TCO Free CEs for DSSCs

Although graphite and graphene are attractive materials due to their good electrical conductivity, thermal stability and high surface area [45,46] it still may possess a limited number of active sites for electrocatalysis [18]. Therefore we used an electrically conductive polymer PEDOT:PSS as an additive to a GNPs film. We expected that the addition of PEDOT:PSS would improve the adhesion of the GNPs to a glass substrate and result in an increase of the electrical conductivity as well as electron transfer in the Pt and FTO free CEs/DSSCs [16] due to the PEDOT:PSS acting as an electrically conductive filler between the conductive GNP sheets and thus reducing potential insulating gaps present between sheets in a pure GNP film. Previously, it was found that an increase in chain length/molecular weight of PEDOT leads to the increase in electron mobility due to the fundamental mechanism of electrical conductivity in polymers [47,48]. Therefore, we used in this work Clevios PH 1000 PEDOT:PSS composition, which has a higher average molecular weight over other grades. Though as-prepared PEDOT:PSS films from PEDOT:PSS aqueous solutions of different grades have almost the same conductivity, typically in the range of 0.1–1 S·cm^−1^, the conductivity of PEDOT:PSS films can be dramatically improved using certain treatments [49]. It is known that the addition of organic solvents with high a boiling point, such as ethylene glycol (EG), Dimethyl sulfoxide (DMSO) and N-Methyl-2-pyrrolidone (NMP) significantly improve the electrical conductivity of PEDOT:PSS by up to 2 or 3 orders of magnitude [50,51,52,53]. Among these organic solvents, NMP has a lower viscosity (1.7 cP) compared to the viscosity of EG (16.9 cP) and DMSO (1.99 cP). Therefore, in this work we used NMP as an additive (10 vol%) to PEDOT:PSS and GNPs dispersions. The addition of NMP to the PEDOT:PSS-GNP mixture also improved the dispersion of the GNPs and as a result the composite film was expected to show enhanced electrical conductivity compared to a pure PEDOT:PSS film.

GNPs of type 1 (surface area of 50 m^2^/g), GNPs type 2 (surface area of 100 m^2^/g) and GNPs type 3 (surface area of 600–750 m^2^/g) have each been added to three separate aqueous PEDOT:PSS dispersions in 10 vol% of NMP and 20 vol% of deionised water (Millipore) and sonicated in closed glass vials for 3 h to produce homogeneous dispersions. Then the three types of GNPs with NMP diluted PEDOT:PSS dispersions were deposited on non-conducting glass substrates by a simple drop casting method. The produced samples have been studied by Raman spectroscopy, Scanning Electron Microscopy (SEM), Thermogravimetric Analysis (TGA) and electrical conductivity measurements.

### 2.2. Raman Spectroscopy Studies

**Figure 1:**
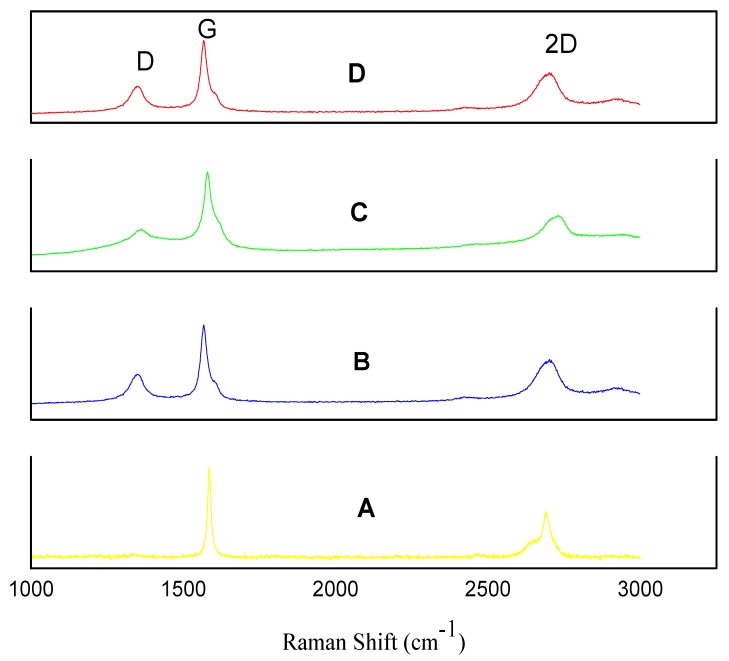
Raman spectra of: (**A**) Graphite powder; (**B**) Graphene nano-platelets (GNPs) type 1 powders; (**C**) GNPs type 2 powders and (**D**) GNPs type 3 powders.

Raman spectroscopy has historically played an important role in the structural characterization of graphitic materials, in particular providing valuable information about defects and stacking of the graphene layers [54]. Initially, Raman spectroscopy of GNPs type 1, GNPs type 2 and GNPs type 3 powders was performed using graphite powders as a comparison (Figure 1). Raman spectroscopy can clearly distinguish single layer graphene from multiple layer graphene and unexfoliated graphite [55]. The main features in graphite/graphene Raman spectra are represented by the D, G and 2D peaks [56]. The graphitic G peak located at ~1580 cm^−1^ and 2D peak located at ~2700 cm^−1^ are always observed in graphite samples [57]. The graphite’s 2D band (~2700 cm^−1^) always has a shoulder at ~2650 cm^−1^ and is representative of graphite [55] as shown Figure 1A. The graphite powders displayed a small D band (~1330 cm^−1^). The D band is indicative of defects or edges in the sample. The ratio of the intensity of the D band, (~1330 cm^−1^) to that of the G band (~1580 cm^−1^) band indicates the structure, size, and defects of the carbon material [31,58,59]. Figure 1A–C shows Raman spectra of GNP type 1, GNP type 2 and GNP type 3 respectively. All three types of GNP powders show a large D peak compared to the D peak of graphite (Figure 1A), which can either be due to defects or most likely because of smaller flakes *i*.*e*., edge effect. ID/IG ratio was measured as shown in Table 1. One would expect that smaller flakes (GNPs type 3) would show a higher ID/IG ratio compared to large flakes (GNPs type 2 and GNPs type 1) [58]. However, the ID/IG ratio of GNPs type 3 powder was (0.40) similar to GNPs type 1 powder (0.40) and a little higher than GNPs type 2 powders (0.34). This suggests that GNPs type 1 and type 2 might have some defect or might have some edge functional groups. The shape of the 2D peak spectra with no shoulder is consistent for all three types of GNPs powder as shown in Figure 1B–D. This indicates the presence of multiple layer graphene flakes in all three types of powders. In summary, the Raman spectroscopy analysis confirmed that all three types of GNPs are multiple layer graphene flakes.

**Table 1:** Intensity ratios for D and G band (calculated from corresponding Raman spectra.

Powder Type	ID/IG
Graphite powder (Reference)	0.02
GNPs type 1	0.40
GNPs type 2	0.34
GNPs type 3	0.40

### 2.3. Scanning Electron Microscopy (SEM) Studies

**Figure 2:**
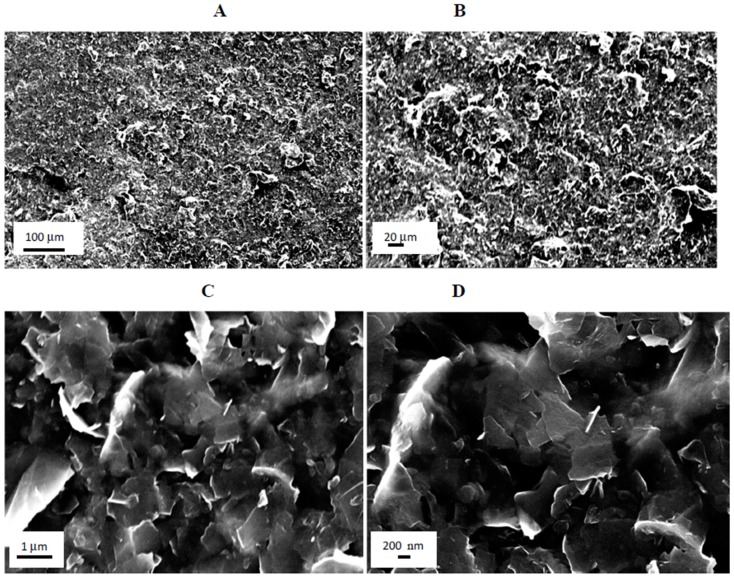
Representative SEM images of GNPs type 2 plus poly (3,4-ethylenedioxythiophene) poly(styrenesulfonate) (PEDOT:PSS) composite film/CE, (**A**,**B**) panels show SEM images of surface; (**C**,**D**) panels shows SEM of fractured edge of the composite film/CE.

SEM was performed only on the samples with the best photovoltaic performance, *i*.*e*., GNPs type 2 with NMP doped PEDOT:PSS composite film/CE. SEM was used to investigate the morphology of the GNPs type 2 plus NMP doped composite film/CE. SEM of the surface as well as a fractured edge of the composite film/CE was also performed. Figure 2 panels A and B display representative SEM images of the surface with panels C and D showing the fracture edge of GNPs type 2 plus NMP doped PEDOT:PSS composite film/CE. In both surface and fracture images of the film, SEM investigation reveals that GNPs were homogeneously dispersed and well interconnected in the composite film/CE.

### 2.4. Thermogravimetric Analysis (TGA) Studies

**Figure 3:**
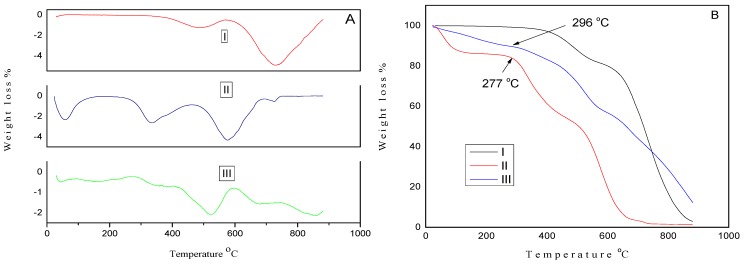
(**A**) Derivative Thermogravimetric Analysis (DTGA) curves of (I) GNPs type 2 powder; (II) pure PEDOT:PSS film and (III) GNPs type 2 + N-Methyl-2-pyrrolidone (NMP) doped PEDOT:PSS composite film/CE; (**B**) TGA curves of (I) GNPs type 2 powder; (II) PEDOT:PSS film and (III) GNPs type2 + NMP doped PEDOT:PSS composite film/CE.

The thermal stability of GNPs type 2 powders, pure PEDOT:PSS film and GNPs type 2 plus NMP doped PEDOT:PSS composite films were also investigated by TGA. Figure 3A displays derivative (DTGA) curves and original TGA curves are presented in Figure 3B. DTGA (Figure 3A(I)) curve of GNP type 2 powder demonstrated an initial weight loss of 14% between 390 and 581 °C, suggesting the removal of some functional groups [60,61,62], further weight loss between 587 and 881 °C can be attributed to the degradation of GNPs. DTGA curve (Figure 3A(III)) of the GNPs type 2 plus NMP doped PEDOT:PSS composite film showed an initial 6 wt% was lost between 30 and 162 °C. This initial weight loss is due to the presence of some organic solvent and water in the film, similarly to one observed in published reports for a PEDOT:PSS film [63,64] and also similar to DTGA of the pure PEDOT:PSS film as shown in Figure 3A(II). The second weight loss (8.4%) between 296 and 419 °C and third weight loss (28.47%) between 419 and 596 °C correspond to the degradation of PEDOT:PSS. Further weight loss of 50 wt% between 607 and 881 °C corresponds to the degradation of GNPs type 2 as DTGA curve (Figure 3A(I)) of pure GNP type 2 powder shows maximum degradation at 733 °C. The TGA curve showed that the GNPs type 2 plus PEDOT:PSS composite film (Figure 3B(III)) is thermally more stable than the PEDOT:PSS dried film (Figure 3B(II)), as degradation of PEDOT:PSS started in GNPs type 2 plus NMP doped composite film at 296 °C which is 19 °C higher than the degradation of the pure PEDOT:PSS film (277 °C) [64].

### 2.5. Electrical Conductivity Measurement

**Figure 4:**
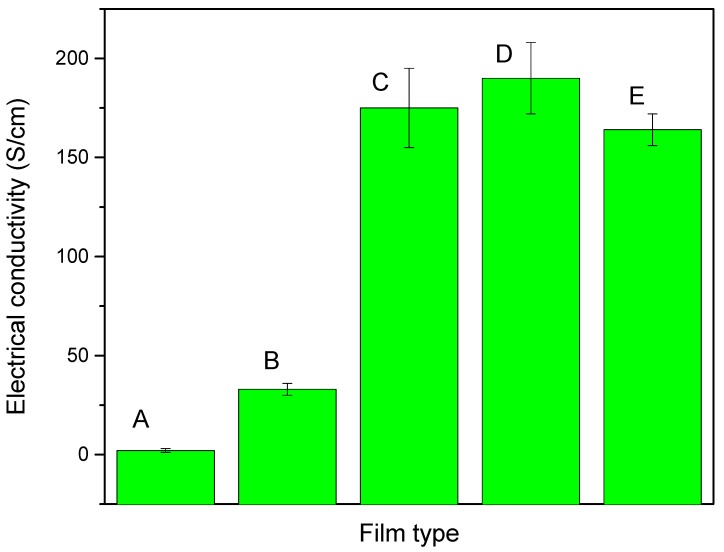
The electrical conductivity of (**A**) pure PEDOT:PSS film; (**B**) NMP doped PEDOT:PSS film; (**C**) GNPs type 1 + NMP doped PEDOT:PSS composite film; (**D**) GNPs type 2 plus NMP doped PEDOT:PSS film and (**E**) GNPs type 3 + NMP doped PEDOT:PSS composite film.

Initially two types of PEDOT:PSS films were prepared and their conductivity was measured: (1) pure PEDOT:PSS film and (2) NMP (10 vol%) doped PEDOT:PSS film. The thickness of the films was in the range of 15 to 20 μm and was measured by digital micrometer caliper. For electrical conductivity measurements, three samples from each type of film were selected and the electrical conductivity was measured and a standard deviation was calculated. The results are presented in Figure 4. The electrical conductivity of the NMP doped PEDOT:PSS film was 33 S/cm an increase of 30.89 S/cm compared to the pure PEDOT:PSS film (2.11 S/cm). The electrical conductivity of the NMP doped PEDOT:PSS film increased by around a factor of 15 compared to the pure PEDOT:PSS film. This electrical conductivity enhancement is attributed to a greater connectivity between the conducting PEDOT chains due to the removal of insulating PSS from the PEDOT:PSS film [65]. Subsequently the electrical conductivity of GNPs type 1, GNPs type 2 and GNPs type 3 plus NMP doped PEDOT:PSS composite films were also measured. The electrical conductivities were dramatically increased by the addition of each type of GNP to the PEDOT:PSS composite films compared to the NMP doped PEDOT:PSS film. However, the highest electrical conductivity of 190 S/cm was observed for the GNP type 2 plus NMP doped PEDOT:PSS composite film. The electrical conductivities of GNPs type1 plus NMP PEDOT:PSS and GNPs type 3 plus PEDOT:PSS composite films were 175 S/cm and 164 S/cm respectively. These results indicate that the addition of all types of GNPs strongly influences the electrical conductivity of NMP doped PEDOT:PSS films.

As it was expected from the Raman spectroscopy investigation, GNPs type 3 powder which has the lowest level of defects should show the highest conductivity and *vice versa* for type 1 and type 2 powders. However, GNPs type 3 plus NMP doped PEDOT:PSS showed the lowest electrical conductivity among the three GNPs types plus PEDOT:PSS composite films. Therefore, these results suggest that electrical conductivity of GNPs plus NMP doped PEDOT:PSS composites films greatly depends on the diameter and surface area of the GNPs used. GNPs type 1 and type 2 have a similar average diameter (up to 5 µm) but different surface areas (GNPs 1 = 50 m^2^/g and GNPs 2 = 100 m^2^/g). Thus we can say that electrical conductivity of GNPs type 2 is greater than GNPs 1 in NMP doped PEDOT:PSS films due to its higher surface area. GNPs type 3 have a very high surface area (600–750 m^2^/g) but shows a lower electrical conductivity (164 S/cm) than the GNPs type 1 and GNPs type 2 plus NMP doped PEDOT:PSS composite films. This is thought to be due to the large contact resistance between small-size GNPs type 3 (diameter = 2 µm) which as a result shows a lower electrical conductivity than GNPs type 1 and GNPs type 2 based films. These results show that large diameter GNPs (GNPs type1 and GNPs type 2) have a higher electrical conductivity than small diameter GNPs type 3 in NMP doped PEDOT:PSS composite films. The conductivity between GNPs 1 and 2 composites is quite close and within experimental error so there is little difference between GNPs 1 and 2 composites and a possible slight increase in the GNPs 2 composite is due to a greater number of flakes and lower flake thickness (less graphene layers in the flake) and thus increasing the connectivity of the flakes and overcoming any negative effect of increased junction resistance.

### 2.6. Investigation of the Photovoltaic Performance of DSSCs

**Figure 5:**
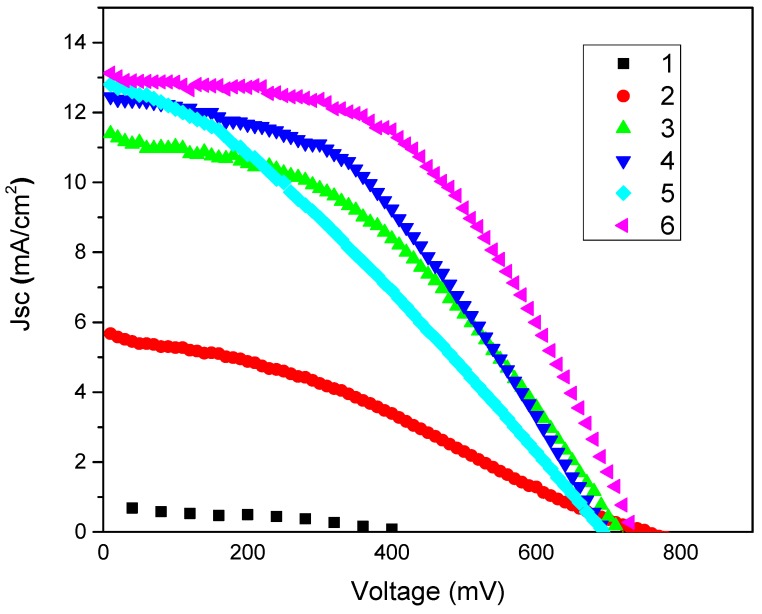
The photovoltaic (J–V) curves of: (1) pure PEDOT:PSS (2) NMP doped PEDOT:PSS; (3) GNPs type 1 + NMP doped PEDOT:PSS; (4) GNPs type 2 + NMP doped PEDOT:PSS; (5) GNPs type 3 + NMP doped PEDOT:PSS and (6) Pt/FTO/CEs-DSSCs.

In this work all DSSCs have been fabricated by using an “open cell approach” (see experimental section for details). Two kinds of reference DSSCs were fabricated: one with a standard Pt/FTO/glass-CE and a second one with a Pt and FTO free CE with NMP doped PEDOT:PSS/glass. Three types of composite films were prepared *i*.*e*., (A) GNPs type 1 plus NMP doped PEDOT:PSS, (B) GNPs type 2 plus NMP doped PEDOT:PSS and (C) GNPs type 3 plus NMP doped PEDOT:PSS and used as Pt and TCO free CEs in DSSCs. The performances of the GNP-PEDOT:PSS composite electrodes were compared with standard Pt/FTO/glass-CE/DSSC under the same conditions. Table 2 summaries the photovoltaic parameters *i*.*e*., open-circuit voltage (Voc), short-circuit current density (Jsc), fill factor (FF), and power conversion efficiencies of all DSSCs which were prepared in this work. The photocurrent density-voltage (J–V) characteristics of the DSSCs with various CEs are shown in Figure 5. The Pt/FTO/glass-CE/DSSC demonstrated a power conversion efficiency of 4.72%. The power conversion efficiency of the NMP doped PEDOT:PSS-CE/DSSC without Pt and FTO was 1.35%. As expected the addition of all three types of GNPs to PEDOT:PSS films resulted in the increase of the power conversion efficiencies of Pt and FTO free CEs-DSSCs. The addition of GNPs type 1 to NMP doped PEDOT:PSS film/CE resulted in an increase of the power conversion efficiencies from 1.35% (NMP doped PEDOOT:PSS/glass-CE) to 3.36%. This power conversion efficiency was improved mainly due to increases in FF from 0.31 to 0.41 and Jsc from 5.67 to 11.4 to mA/cm^2^. GNPs type 2 plus NMP doped PEDOT:PSS CE also improved the power conversion efficiencies from 1.35% (NMP doped PEDOOT:PSS/glass-CE) to 3.70%. Again the improvement of power conversion efficiencies of GNPs type 2 with NMP doped PEDOT:PSS was mainly due to the increase in FF from 0.31 to 0.43 and Jsc from 5.67 mA/cm^2^ to 12.45 mA/cm^2^. The addition of GNPs type 3 to NMP doped PEDOT:PSS film/CE improved the power conversion efficiencies from 1.35% (NMP doped PEDOOT:PSS/glass-CE) to 2.78%. The power conversion efficiencies of GNPs type 3 with NMP doped PEDOT:PSS was mainly increased due to the rise in Jsc from 5.67 to 12.80 mA/cm^2^ while FF (0.33) was similar to the NMP doped PEDOT:PSS film/glass-CE/DSSC. These results demonstrate that the addition of all three types of GNPs to NMP doped PEDOT:PSS in general resulted in an increase of the electrocatalytic activity of the CE as the Jsc was increased in all cases. However, the GNPs type 1 and type 2 plus NMP doped PEDOT:PSS composite films/CEs showed an increase in FF as well as in Jsc. This suggests that these two composite films/CEs are more conductive than the GNPs type 3 plus NMP doped PEDOT:PSS composite film/CE and the NMP doped PEDOT:PSS/CE. This was also confirmed and discussed in the Section 2.5.

**Table 2:** The photovoltaic parameters of the Pt and FTO free CEs-DSSCs with various types of GNPs plus PEDOT:PSS composite films (measured at 100 mW/cm^2^; cell active area was 0.8 cm^2^).

CE Type	Voc (V)	Jsc (mA/cm^2^)	FF	Efficiency (%)
Pt/FTO/glass	0.730	13.12	0.49	4.72 ± 0.10
NMP doped PEDOT:PSS/glass/CE	0.760	5.67	0.31	1.35 ± 0.05
GNPs type 1 + NMP doped PEDOT:PSS/glass	0.710	11.4	0.41	3.36 ± 0.06
GNPs type 2+NMP doped PEDOT:PSS/glass	0.690	12.45	0.43	3.70 ± 0.10
GNPs type 3 + NMP doped PEDOT:PSS/glass	0.690	12.80	0.31	2.78 ± 0.08

Overall the power conversion efficiencies have the following trend: Pt/FTO/glass > GNPs type 2 plus NMP doped PEDOT:PSS/glass > GNPs type 1 plus NMP doped PEDOT:PSS/glass > GNPs type 3 plus NMP doped PEDOT:PSS/glass > NMP doped PEDOT:PSS/glass. The two main factors that are responsible for improving power conversion efficiencies are the electrical conductivity and electrocatalytic activity of the CE. The highest Jsc value (13.12 mA/cm^2^) and FF (0.49) was obtained with Pt/FTO/glass-CE/DSSC. This shows that the Pt/FTO/glass-CE is more electrically conductive and more electrocatalytically active than the GNP type 2 plus NMP doped PEDOT:PSS composite films/CEs. In the case of the GNPs filled samples, type 2 and 1 plus NMP doped PEDOT:PSS composite film/glass-CE have power conversion efficiencies of 3.70% and 3.36% respectively The fact that cells with GNP type 2 have a higher power conversion efficiency is most likely due to the higher surface area of type 2 GNPs that results in higher electrical conductivity and higher electrocatalytic activity.

In general, graphitic materials, such as CNTs and graphite, have basal and edge planes. Basal planes exhibit slow electron transport, whereas edge planes exhibit fast electron transport [61,66]. Among these three GNPs type the GNPs type 3 has the highest surface area, which means GNP type 3 might have many edge planes or surfaces available for reduction of the tri-iodide ion in electrolytes. Therefore, it was expected that GNP type 3 with NMP doped PEDOT:PSS composite film/CE should be more electrocatalytically active than GNP type 1 and GNP type 2 with NMP doped PEDOT:PSS composite/glass- CE/DSSC. The lower power conversion efficiency of the DSSC with GNPs type 3 with NMP doped PEDOT:PSS composite film/CE could be due to large contact resistances caused by the involvement of a large number of small size GNPs per unit volume compared to GNP of type 1 and type 2. This large contact resistance per unit volume can have a negative effect on the electrical transport properties of GNP type 3 based films/CEs and result in lower electrical conductivity hence lower power conversion efficiency than GNP type 1 and GNP type 2. Overall the addition of all three types of GNPs into NMP doped PEDOT:PSS CE/DSSC did not significantly affect the Voc in comparison to standard Pt/FTO/glass-CE/DSSCs. The standard Pt/FTO/glass-CE shows Voc of 0.730 mV while, NMP doped PEDOT:PSS, GNPs type 1 with NMP doped PEDOT:PSS composite/glass-CE, GNPs type 2 with NMP doped PEDOT:PSS composite/glass-CE and GNP type 3 with NMP doped PEDOT:PSS composite/glass-CE/DSSCs have shown Voc of 0.760, 0.710, 0.690 and 0.690 respectively.

### 2.7. Investigation of Sintering on the Properties of the CEs and Performance of the Corresponding DSSCs

#### 2.7.1. Effect of Sintering on the Electrical Conductivity of GNPs Type 2 with NMP Doped PEDOT:PSS Films/CEs

**Figure 6:**
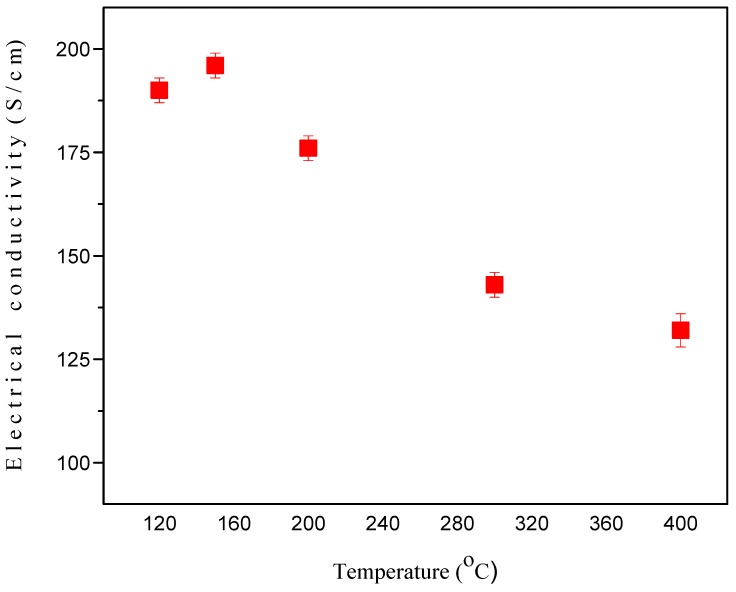
The electrical conductivity of GNPs type 2 plus PEDOT:PSS composite film/CE which was sintered at 120 °C, 150 °C, 200 °C, 300 °C and 400 °C.

The electrical conductivity of GNPs type 2 with NMP doped PEDOT:PSS composite films/CEs, which were sintered at: 120 °C, 150 °C, 200 °C, 300 °C and 400 °C for 20 minutes were measured. The results are presented in Figure 6. The electrical conductivity of GNPs type 2 with NMP doped PEDOT:PSS composite film/CE which was sintered at 120 °C is 190 S/cm. The electrical conductivity increased to 196 S/cm for GNPs type 2 plus NMP doped PEDOT:PSS composite films/CEs which were sintered at 150 °C. However electrical conductivity of the sample decreased to 176 S/cm as it was sintered temperature up to 200 °C. Then the electrical conductivity further decreased to 143 S/cm and 132 S/cm with a sintered temperature of 300 °C and 400 °C respectively. This shows that PEDOT:PSS starts decomposing in the temperature range of 200 to 400 °C which is consistent with TGA data. In summary, the electrical conductivity investigation demonstrated that 150 °C is an optimal sintering temperature for GNPs type 2 plus NMP doped composite film/CE at which GNPs are more highly interconnected in the composite film and that results in the efficient electron transfer from one GNP to another and hence an increase in electrical conductivity.

#### 2.7.2. Raman Spectroscopy of GNPs Type 2 with NMP Doped PEDOT:PSS Films/CEs Sintered at Various Temperatures

**Figure 7:**
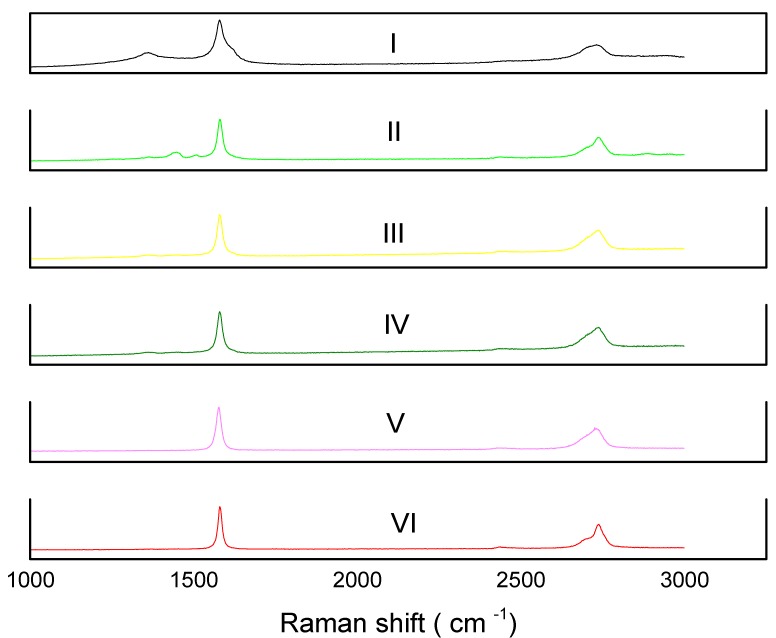
(I) Raman Spectra of, (I–VI) shows GNPs type 2 + PEDOT:PSS composite films/CEs, sintered at 120 °C, 150 °C, 200 °C 300 °C and 400 °C respectively.

Raman spectroscopy was employed to investigate a change in the graphitic structure during the sintering process of GNPs type 2 plus NMP doped PEDOT:PSS composite films/CEs as shown in Figure 7. The defectiveness in graphite samples are generally defined by the ratio of the intensity of the defect band (D band, ~1300 cm^−1^) to that of the G band (~1580·cm^−1^), ID/IG. Table 3 shows the ID/IG ratio calculated for the sintered films. ID/IG for GNP type 2 was 0.34. The ID/IG was decreased to 0.13 for GNPs type 2 plus NMP doped PEDOT:PSS composite film/CE which was sintered at 120 °C. The ID/IG ratio of GNPs plus NMP doped PEDOT:PSS composite films/CEs which were sintered at 150 °C, 200 °C, 300 °C and 400 °C were 0.13, 0.13, 0.02 and 0.06 respectively. These results suggest that as supplied GNPs type 2 are partially oxidised/functionalised graphene *i*.*e*., that have some edge functional groups. The sintering process reduces the attached functional groups and thus results in a decrease of the ID/IG for GNPs type 2 in the composite film/CE. In summary, Raman studies indicate that electrical conductivity hence power conversion efficiency can be further improved if the initial GNPs powder is pre-sintered at 300 °C.

#### 2.7.3. Effect of Sintering Temperature on the Photovoltaic Performance of DSSCs with GNPs Type 2 with PEDOT:PSS Composite Films/CEs

**Figure 8:**
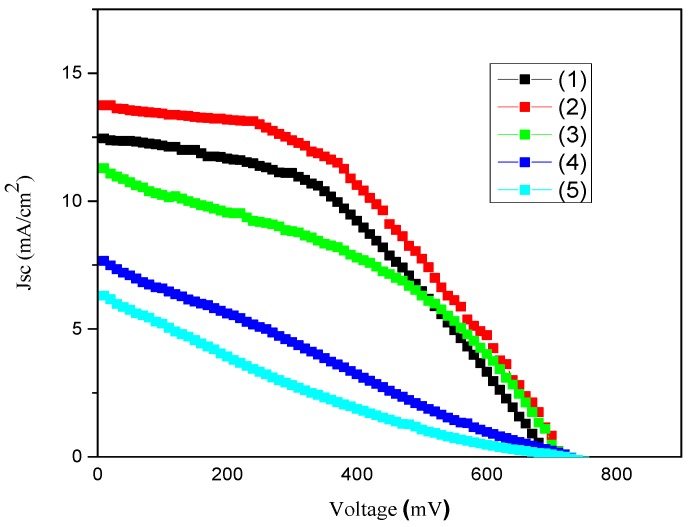
The photovoltaic (J–V) curves of GNPs type 2 + NMP doped PEDOT:PSS film/CE-DSSCs which were sintered at (1) 120 °C; (2) 150 °C; (3) 200 °C; (4) 300 °C; (5) 400 °C.

GNPs type 2 plus NMP doped PEDOT:PSS composite films gave the best power conversion efficiency and higher electrical conductivity in comparison to GNP type 1 and GNPs type 3 plus NMP doped PEDOT:PSS composite films. Therefore for further investigation the GNPs type 2 plus PEDOT:PSS composite film/CE was selected. GNP type 2 plus PEDOT:PSS composite films were sintered at various temperatures for 20 min as shown in Table 4 and the photocurrent density-voltage (J–V) characteristics of the DSSCs are shown in Figure 8. The purpose of this investigation was to ascertain the optimal sintering temperature at which the GNPs type 2 plus PEDOT composite film/CE has the best performance. The highest power conversion efficiency of 4.29% was observed for the CE which was sintered at 150 °C. The power conversion efficiency of GNPs type 2 with NMP doped composite film/CEs sintered at 200 °C decreased to 3.23%. However, power conversion efficiencies were dramatically decreased to 1.35% and 0.84% for GNP2/PEDOT:PSS/CEs which were sintered at 300 °C to 400 °C respectively. These results show that GNPs type 2 and NMP doped PEDOT:PSS film/CE was fully dried at 150 °C and PEDOT:PSS degradation starts at a higher sintering temperature (274 °C to 400 °C). Therefore, the power conversion efficiencies were decreased at high temperature sintering as this was confirmed by the above TGA study.

**Table 3:** Intensity ratios for D and G band (calculated from corresponding Raman spectra.

Sintering Temperature	ID/IG
GNPs 2 type powder	0.34
120 °C	0.13
150 °C	0.13
200 °C	0.13
300 °C	0.02
400 °C	0.06

**Table 4:** The effect of sintering on the photovoltaic performance of the DSSCs with GNPs type 2 and NMP doped PEDOT:PSS hybrid CEs. Measured at 100 mW/cm^2^, cell active area: 0.8 cm^2^.

CE Type	Sintering Temp. (°C)	Voc (V)	Jsc (mA/cm)	FF	Efficiency (%)
GNPs 2 + NMP doped PEDOT:PSS/CE	120	0.690	12.45	0.43	3.70 ± 0.10
As above	150	0.710	13.75	0.44	4.29 ± 0.12
As above	200	0.710	11.28	0.40	3.23 ± 0.09
As above	300	0.720	7.66	0.24	1.35 ± 0.08
As above	400	0.720	6.03	0.18	0.84 ± 0.05

These results demonstrate that the optimum temperature for sintering of GNP 2 plus NMP doped PEDOT:PSS is 150 °C. The Jsc value of the GNPs type 2 plus NMP doped PEDOT:PSS composite film/CE was also initially improved from 12.45 (sintered at 120 °C) to 13.75 mA/cm^2^ (sintered at 150 °C) but the Jsc values were linearly decreased to 11.28 mA/cm^2^ (sintered at 200 °C), 7.66 mA/cm^2^ (sintered at 300 °C) and 6.3 mA/cm^2^ (sintered at 400 °C). Voc values were not significantly affected by sintering temperature, the Voc values of 0.690 V, 0.710 V, 0.710 V, 0.720 and 0.720 V were observed for GNPs type 2 plus NMP composite film/CEs which were sintered at 120 °C, 150 °C, 200 °C, 300 °C and 400 °C respectively. The FF values (0.43) initially remained stable for GNPs type 2 plus NMP doped composite films/CEs which were sintered at 120 °C and 150 °C but FF values were greatly decreased to 0.40, 0.22 and 0.18 for CEs which were sintered at 200 °C, 300 °C and 400 °C respectively. This reveals that the internal resistance of the CE/DSSC was increased at high sintering temperature due the degradation of PEDOT:PSS in the composite film/CE, therefore the FF values decreased accordingly. This demonstrated the importance of PEDOT:PSS in GNPs type 2 composite films/CEs and that PEDOT is well adhered on the surfaces of the GNPs, therefore the GNPs are highly interconnected and as a result the electrons are efficiently transferred from one GNP to another which also results in an increase in the electrocatalytic activity of the composite film/CE. Previously, using cyclic voltammetry it was demonstrated that reduced graphene oxide based and graphene/polyimide based electrodes can provide many catalytic sites for charge-transfer, particularly on the numerous edges of graphene sheets. As a result these electrodes possess a higher current in the reduction of I_3_^−^ and corresponding DSSCs demonstrate a comparable conversion efficiency to the conventional sputter-Pt/FTO CE containing DSSCs [67,68]. We believe that our GNP based electrodes demonstrate similar behavior.

## 3. Experimental Section

### 3.1. General Procedures

Organic solvent based electrolytes (High Performance Electrolyte), Thermoplastic sealant (25 µm) and dye (N-719) were purchased from Dyesol Ltd., Queanbeyan, Australia. Graphene nanoplatelets (GNPs) type 1, type 2 and type 3 were purchased from Cheap Tubes Inc., Cambridgeport, VT, USA (www.cheaptubesinc.com), with surface area: GNPs type 1 = 50 m^2^/g, GNPs 2 = 100 m^2^/g and GNPs type 3 = 600–750 m^2^/g, and diameter: GNPs 1 around 5 µm, GNPs 2 around 5 µm and GNPs 3 around 2 µm). PEDOT:PSS aqueous dispersion (Clevios PH 1000) was purchased from Heraeus Clevios GmbH (Leverkusen, Germany). The weight ratio of PSS to PEDOT in Clevios PH 1000 is 2.5; the average molecular weights of PSS and PEDOT are about 400,000 g/mol and 1000–2500 g/mol respectively. Non-conducting glass was purchased Sigma Aldrich. Screen printed working electrodes (TiO_2_ layer with ~14 µm thickness), counter electrode (Pt coated) were purchased from Solarprint Ltd. (Dublin, Ireland).

Ultrasonic Bath Grant XB6 (200 W) was used in all sonication experiments. Raman spectra (excitation at 633 nm) were recorded on a Horiba Jobin Yvon LabRAM-HR (100× objective lens). SEM images of dry film have been taken using Zeiss Ultra plus SEM. The electron beam generator is a thermal field emission tungsten tip, with a sintered reservoir of zirconium oxide in the shank. For these samples, the electrons are accelerated between 6 and 8 kV. Thermogravimetric analysis was done using Perkin Elmer Pyris 1 TGA. TGA experiments were carried out under oxygen atmospheres. Each of the samples was heated from 30 to 900 °C at the rate of 10 °C/min. Measurements of I–V curves were performed using a digital source meter (Keithley 2400, Cleveland, OH, USA) and a standard solar simulator (Oriel 92193, Oriel GmbH, Darmstadt, Germany), which was equipped with a 1.5 G air mass filter and calibrated with a silicon-based reference cell. Electrical conductivity measurements were made by using a source meter (Kiethley 2004, Cleveland, OH, USA). which was interfaced with a computer using I–V 0001 software. For electrical conductivity measurements, three samples from each type of film were selected and then the electrical conductivity was measured and a standard deviation was calculated.

### 3.2. Preparation of Pt and TCO Free CEs for DSSCs

The three aqueous PEDOT:PSS dispersions were diluted with 10 vol% of NMP and 20 vol% of deionised water (Millipore, Darmstadt, Germany) and sonicated in an ultrasonic bath Grant XB6 (200 W) for 30 min. This was then split into three equal parts and GNPs type 1, GNPs type 2, GNPs type 3 were added to each part at 66 wt% of solid PEDOT:PSS content, to make three sample dispersions. Each sample was sonicated in closed glass vials for 3 h in an ultrasonic bath Grant XB6 (200 W) to make homogeneous dispersions. To prepare CEs for the above dispersions a non-conducting glass (NCG) substrate was used. The NCG was cleaned by ultrasonication in deionised water (Millipore, Darmstadt, Germany) for 20 min and then sonicated in ethanol for a further 20 min. This was followed by heating in an oven at 80 °C to remove any residual solvent traces. After cleaning, adhesive tape was applied on all four edges of the NCG substrate. Various samples of pre-prepared dispersions were drop casted on the NCG substrate using a pipette. Each sample was then covered with a petri dish and left for 2 h at room temperature to stabilize. The GNP based CEs were then dried by heating at 80 °C in an oven for 40 min and finally heated at 120 °C for a further 20 min to remove any residual solvent. The thickness of the composite film was controlled by squash tape or volume dropped. The thicknesses of these films were in the range of 15 µm to 20 µm measured by SEM cross sectional characterization. The DSSCs with these various GNPs based CEs and reference CE with standard Pt/FTO/glass were fabricated with TiO_2_ coated electrodes/WEs which were sintered at 450 °C in an oxygen atmosphere in a furnace and allowed to cool to 80 °C, then soaked in 3 × 10^−4^ M solution of N719 (dye) in ethanol in a closed container at room temperature for 24 h. After dye adsorption the original white TiO_2_ film became reddish. Dyed TiO_2_ films were washed with ethanol to remove the excess dye which is not firmly adsorbed to the surfaces of the TiO_2_. Then a 25 µm thick surlyn spacer was coated around an uncoated TiO_2_ film and the composite films of GNPs and PEDOT:PSS were tested in DSSCs as Pt and TCO free CEs.

## 4. Conclusions

In this work we have prepared and investigated new Pt and TCO free counter electrodes with three different types of graphene nano-platelets (GNPs), which were dispersed into a NMP doped/diluted PEDOT:PSS/H_2_O matrix and deposited onto a non-conducting glass substrate. In this case GNP based composite materials acted as an electrocatalyst for I_3_^−^ reduction and charge transport material in a Pt and TCO free CE based DSSC. Initially, the highest power conversion efficiency of 3.70% was achieved for DSSCs with GNPs type 2 with NMP doped PEDOT:PSS composite film/glass-CE compared with the power conversion efficiencies of 1.35% for NMP doped PEDOT:PSS film/glass-CE. Thus the power conversion efficiency of GNPs type 2 with NMP doped PEDOT:PSS composite film-CE was increased by a factor of 2.27 compared to the only NMP doped PEDOT:PSS film/glass-CE/DSSC mostly due to the improvement in the electrical conductivity of the composite film. However, the power conversion efficiency of GNPs type 2 with NMP doped PEDOT:PSS composite film/CE was still ~1% lower than standard Pt/FTO/glass-CE/DSSC (4.72%). While in the case of GNPs type 1 and type 3 plus NMP doped PEDOT:PSS composite film-CEs the power conversion efficiencies also improved from 1.35% to 3.36% and 2.78% respectively, which is an increase by a factor of 2.48 for (GNPs type 1) and 2.05 for (GNPs type 3) compared to a power conversion efficiency of 1.35% for NMP doped PEDOT:PSS film/glass-CE/DSSC. An electrical conductivity study of the composites revealed that the addition of all three types of GNPs increased the electrical conductivity of NMP doped PEDOT:PSS composite films/CEs but GNPs type 2 plus PEDOT:PSS composite film/CE showed the highest conductivity of 190 S/cm.

Most importantly, it was found that the power conversion efficiency can be further increased up to 4.29% by optimizing the sintering temperature for GNPs type 2 plus NMP doped PEDOT:PSS composite CE. Electrical conductivity studies demonstrated that the effect of sintering temperature on the GNPs type 2 plus NMP doped PEDOT:PSS composite films/CEs caused an initial increase in conductivity before falling off at higher sintering temperatures with the highest conductivity (196 S/cm) achieved at an annealing temperature of 150 °C. The thermal stability of the GNPs type 2 with NMP doped PEDOT:PSS, GNPs type 2 powders and pure NMP doped PEDOT:PSS was also investigated by TGA which showed that GNPs type 2 plus PEDOT:PSS composite film is more stable than the pure PEDOT:PSS film. The Raman spectroscopy studies demonstrated that supplied GNPs type 2 contained a partially oxidised/functionalised graphene *i*.*e*., they have some edge functional groups. Therefore, the ID/IG ratio decreased at a high sintering temperature as an ID/IG ratio of 0.02 and 0.06 were observed for GNPs type 2 plus PEDOT:PSS composite films which were sintered at 300 °C and 400 °C respectively. Finally, we must notice that in our work these values of efficiencies (up to 4.29% using Pt and TCO free CE) have been achieved for DSSCs, which were produced using an “open cell” method. We had to use this approach due to technical limitations of our fabrication facilities. The “open cell” approach normally results in solar cells with relatively low efficiency. Therefore, we believe that further optimization of the GNP based hybrid CE and corresponding photovoltaic cell fabrication will result in much higher power conversion efficiencies. Also further detailed electrocatalytic activity testing and studies of our electrodes including cyclic voltammetry, computer modelling and other methods will be necessary to perform in the future in order to fully understand the mechanism of the charge transport in these systems.
